# Network Traffic Data Augmentation Using WGAN Model Guided by LLM

**DOI:** 10.3390/s25247457

**Published:** 2025-12-08

**Authors:** Jumanah Hmoud Alyoubi, Miada Almasre, Aishah Aseeri, Alanoud Subahi, Norah Al-Malki

**Affiliations:** 1 Information Technology Department, Faculty of Computing and Information Technology, King Abdulaziz University, Jeddah 21589, Saudi Arabia; malmasre@kau.edu.sa (M.A.); aaaseeri@kau.edu.sa (A.A.); asubahi@kau.edu.sa (A.S.); 2 Department of European Languages, King Abdulaziz University, Jeddah 21589, Saudi Arabia; nasalmalki2@kau.edu.sa

**Keywords:** IoT security, IoT devices identification, network traffic classification, WGAN, LLM, ML

## Abstract

The Internet of Things (IoT) continues to expand across critical infrastructures, enabling automation, efficiency, and data driven decision making; yet, reliable device identification from network traffic remains hampered by severe class imbalance that skews learning and degrades performance. Synthetic data generation offers a promising remedy, particularly in privacy-sensitive security settings where access to representative traffic is limited. This paper advances the state of the art by proposing a framework that unites graph-conditioned generative modeling with large language model (LLM) guidance to produce realistic, semantically valid synthetic network traffic for imbalanced classification. First, we construct feature relationship graphs derived from Pearson correlation, Spearman rank correlation, and mutual information to capture inter-feature dependencies, and use these graphs to condition a Wasserstein GAN (WGAN), thereby preserving structural properties of real traffic during generation. Second, we employ an LLM to define class-specific semantic constraints, including admissible feature ranges, attribute correlations, and protocol level rules, which are enforced as soft guidance to steer the generator toward label-consistent and standards-compliant samples. Third, we institute a dual validation loop that combines LLM-based feedback on constraint satisfaction with evaluation of classifiers trained on datasets balanced by our method versus the traditional SMOTE technique. Lastly, extensive experiments demonstrate that jointly leveraging structural (graph) and semantic (LLM) conditioning yields higher-fidelity synthetic traffic and delivers consistent gains in macro-F1 and balanced accuracy for network traffic classification, highlighting the framework’s utility for security analytics under data scarcity and privacy constraints.

## 1. Introduction

The Internet of Things (IoT) is one of today’s most popular technologies. It spans everything from the smartphones in our pockets to the sensors deployed in industrial factories [1]. By 2025, it is expected that there will be more than 75 billion IoT devices worldwide [2]. In general, IoT refers to large numbers of heterogeneous devices connected to the network typically via gateways that can communicate with each other and take appropriate actions based on the data they collect, analyze, and share. These devices can take appropriate action based on the collected, analyzed, and shared data.

**Figure 1:**
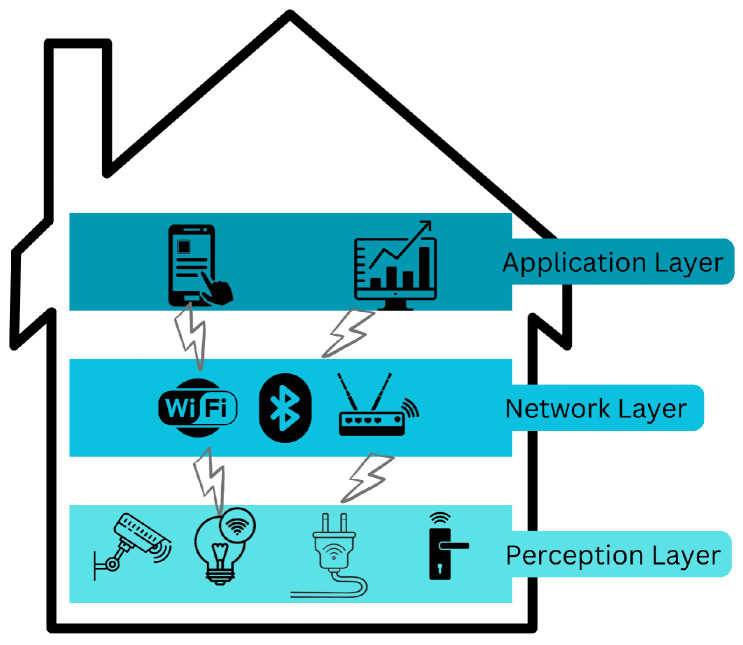
Three-layer IoT architecture.

IoT in smart homes emphasizes on communication between individual nodes that collect and transmit sensitive data and control critical infrastructure. It functions on the three layers presented in Figure 1. Using sensors and actuators, the Perception layer gathers and interprets data from the environment. The network layer uses technologies such as WiFi, LTE, Bluetooth, 3G, and Zigbee to transport and deliver data to IoT hubs and devices via the Internet. The Application layer protects the authenticity, integrity, and secrecy of data in order to achieve the goal of establishing a smart environment. Each layer contains security flaws.

In our work, we focus on network layer and how to protect the smart home network from intruders and eavesdroppers. Ensuring security and privacy requires authenticating the nodes that communicate with each other or with gateways [3]. However, due to the characteristics of these nodes which include limits in terms of processing power and resources and heterogeneity of the connected nodes, the security of IoT is considered challenging.

One of the primary goals in the context of IoT security is to keep essential assets safe from unauthorized access and ensure the integrity of data and systems by verifying the identity of entities trying to access resources or perform actions. Traditional node identifications that rely on MAC addresses, IP addresses, Bluetooth ID, and Zigbee ID may be easily forged [4]. The use of cryptographic techniques for identification and authentication is an effective way to ensure confidentiality and authentication. However, in IoT devices, the vulnerability arises from the use of smaller-bit-sized cryptographic keys due to limited capabilities, making them more susceptible to hacking [5]. Identification of devices within the network plays an important role in preventing unauthorized access to or misuse of network resources. Since the early days of the Internet, network traffic categorization has been a key issue, with numerous methodologies ranging from port-based to statistical and behavioral methods. Internet Service Providers (ISPs) have prioritized network traffic classification in order to manage network performance and security [6]. Because of the significance of privacy and security for providers and end users, this has gained traction in IoT through integration with machine learning. Traffic classification technology has been extensively employed in numerous scholarly investigations within IoT systems [7].

All supervised [3,8], unsupervised [5], and deep learning [9,10] methods have been used for classification and identification. Supervised machine learning showed high accuracy when identifying devices. On the other hand, they encountered various challenges [11], which can be succinctly summarized as follows: must train the model again when a new device type is connected to or added to the network, or when the behavior of the device changes legitimately. Also, to avoid overfitting when training the model, a huge number of labeled and balanced datasets are required. Moreover, detecting unknown traffic is critical for identifying new types of devices and traffic, which cannot be recognized by supervised learning methods.

Due to the limited availability of publicly accessible IoT network traffic datasets, researchers face significant challenges in training and evaluating device identification models. Deploying physical IoT devices to collect real traffic is possible but often costly and difficult to scale to networks with hundreds or thousands of heterogeneous devices, while privacy and confidentiality concerns further restrict the sharing of raw captures [12]. In addition, class imbalance is a well-recognized challenge in IoT device identification: traffic from popular or always on devices (such as cameras or hubs) heavily dominates that of less frequently active or niche devices (such as specific sensors or appliances). This skewed distribution biases classifiers toward majority device types and leads to poor generalization and low identification accuracy for minority devices, even though these rare devices may be operationally important [10].

Traditional oversampling methods such as SMOTE attempt to mitigate class imbalance by interpolating synthetic minority samples in the feature space [13]. However, recent work has shown that SMOTE and its variants can fail to capture complex, non-linear and multimodal distributions; may ignore local density structure; and can place synthetic samples in low-density or overlapping regions, which degrades classifier performance in high-dimensional and noisy settings [13]. As an alternative, generative models based on Generative Adversarial Networks (GANs) have gained traction for data augmentation in cybersecurity and network traffic modelling, including applications to class-imbalanced traffic datasets [14]. While these approaches can better approximate high-dimensional distributions, many GAN-based traffic generators still treat flows as flat feature vectors and do not explicitly encode protocol rules, device-specific behavior, or higher order dependencies between features, which may result in synthetic traffic that matches marginal statistics but lacks semantic consistency and protocol fidelity [15].

Within the family of generative models, Wasserstein Generative Adversarial Networks (WGANs) have been shown to improve training stability and sample quality by optimising the Wasserstein distance between real and generated distributions [16]. Nevertheless, conventional WGAN variants still lack mechanisms to integrate domain-specific knowledge and complex structural dependencies between network traffic features, which can lead to synthetic samples that do not fully respect protocol constraints or realistic IoT device behavior. Recent IIoT traffic fingerprinting studies also report a clear distribution gap between synthetic and real Internet-facing deployments, especially in terms of timing and flow dynamics [17]. Our generative model partly addresses this by being constrained with statistics and semantic rules extracted from real IoT traffic.

To address these challenges, this research presents an integrated framework that combines graph-conditioned WGANs with dynamic guidance derived from LLMs to generate synthetic IoT network traffic that is both realistic and semantically contextual. The proposed framework relies on a feature relationship graph, constructed using a combination of Pearson and Spearman correlation coefficients along with mutual information, to capture meaningful structural dependencies and guide the generation process. In addition, LLMs are used to derive class-specific semantic constraints, including numeric ranges, attribute associations, and protocol rules, thereby improving the plausibility and interpretability of the generated samples. An LLM-based validation mechanism is further employed to assess the reasonableness of the synthetic traffic and its relevance to practical IoT scenarios.

Our investigation offers contributions to both IoT device identification and techniques for handling imbalanced datasets. The main contributions of this work are summarized as follows:

- It presents a comprehensive analysis of machine learning-based approaches for identifying IoT devices using network traffic features.
- It proposes a novel data balancing framework based on Wasserstein Generative Adversarial Networks (WGANs) to generate realistic synthetic samples for underrepresented device classes.
- It demonstrates the effectiveness of the WGAN-based approach in improving classification performance through extensive experimentation and comparison with traditional balancing techniques such as SMOTE.

The remainder of this paper is structured as follows: Section 2 presents a review of the related literature. Section 3 describes the materials and methods employed in this study. The experimental results are detailed in Section 4, followed by an in-depth discussion in Section 5. Finally, Section 6 concludes the paper.

## 2. Literature Review

IoT network traffic has been extensively studied both for analyzing device behavior and for generating realistic synthetic network traffic features. On the analysis side, previous work has explored the characteristics of packet-level and flow-level features, protocol fingerprints, and temporal communication patterns to characterize devices, detect anomalies, and support automated identification. In parallel, a growing line of research investigates data driven generative models for IoT network traffic, which aim to synthesize flows that preserve the statistical and behavioral properties of real deployments. Against this background, the following subsections first review methods for the identification of IoT devices based on network traffic Section 2.1, and then discuss approaches to the generation of IoT traffic Section 2.2.

### 2.1. Methods for IoT Identification

Recent work has systematically reviewed network traffic-based methods for IoT device identification and fingerprinting, organizing existing approaches into rule-based, classical machine learning, and deep learning families, and distinguishing between packet level, flow level, and application/business level fingerprints [17]. This subsection presents a comparative review of various studies on IoT device identification, focusing on approaches based on machine learning and deep learning. It highlights the methodologies, key findings, and advancements in leveraging ML/DL techniques for accurately identifying IoT devices within network environments. It is summarized in the Table 1.

Hamad et al. [3] proposed a passive behavioral fingerprinting technique that uses supervised machine learning on short packet sequences to identify IoT device types and detect unknown or rogue devices, but their method struggles to distinguish similar devices from the same vendor (e.g., D-LinkSensor, D-LinkSiren, and D-LinkWaterSensor). Salman et al. [4] analyzed statistical flow features to classify IoT vs. non-IoT traffic and identify device types, achieving up to 94.5% accuracy for device identification and 97% for abnormal traffic detection using Random Forest; however, the model may not generalize well to unseen device types, traffic patterns, or attacks. Mahmoud et al. [18] introduced a multi step framework that monitors traffic, builds sensor profiles, analyzes communication patterns, and classifies irregularities to enhance IoT device identification and threat mitigation, but their evaluation is limited to only three devices. Song et al. [19] improved discrimination between similar devices from the same manufacturer by extracting packet and sequence level features and proposing the TSMC-SVM multi-class classifier, achieving an average identification accuracy of 93.2%.

**Table 1:** ML algorithms and evaluation metrics in the literature.

Ref.	Class Imbalance Handling	Algorithm	Performance
[20]	One-class classifier on normal traffic	ALOC (1D-CNN DAE + adversarial learning)	Best Acc/Rec/F1; FPR 0.166%, FNR 0.014%
[21]	Traffic diagnosis + normal-only training (no explicit resampling)	Transformer-based two-stage model with result ensemble	Acc up to 100% (with ensemble)
[22]	SMOTE, undersampling, Balance Cascade	Random Forest	Precision 96% Rec 94% F1= 95%
[19]	No sampling; overlap resolved via cosine similarity	TSMC-SVM	Acc 93.2%
[3]	No explicit handling; threshold tuning & F1/α	Random Forest	F1 91%
[18]	Not directly addressed; balanced subset	Random Forest	Accu 100%
[4]	Random oversampling	Random Forest	Acc 94%
[8]	Balanced instance generation	Random Forest	acc 99% Precision 96% Rec 97%
[5]	Not directly handled; feature weighting by variance	Clustering	Not mentioned
[23]	Capped samples per device + threshold tuning	Unsupervised ML	Acc range 80%
[24]	Labeled class upsampling + CCLP loss	CNN	Acc 99%
[9]	Not addressed; imbalance noted	CNN	Acc, Rec, Precision, and f1 = 99%
[10]	Not traditional; Dirichlet priors + 1-class SVM	LSTM	average F1 82% Acc 70%

Mahmoud et al. [18] introduced a multi step framework that monitors traffic, builds sensor profiles, analyzes communication patterns, and classifies irregularities to enhance IoT device identification and threat mitigation, but their evaluation is limited to only three devices. Song et al. [19] improved discrimination between similar devices from the same manufacturer by extracting packet and sequence level features and proposing the TSMC-SVM multi-class classifier, achieving an average identification accuracy of 93.2%. Chowdhury et al. [8] used a device fingerprinting (DFP) technique that filters traffic by MAC address and derives statistical profiles from tcp.window_size and ip.len to train a supervised classifier, yet their accuracy decreases when devices share the same manufacturer and category. Pinheiro et al. [22] relied on simple statistics (mean, standard deviation, and transmitted bytes in one second windows) without payload inspection to classify devices and events in a smart home setting, where Random Forest reached up to 96% accuracy on the UNSW dataset [11].

Nguyen et al. [25] computed entropy-based traffic features and applied RF to identify devices in smart homes with up to 94% accuracy and the ability to flag abnormal behavior, but they did not compare their approach against other recent methods. Noguchi et al. [5] analyzed packet level communication features to distinguish device types and models, assuming that each device exhibits a distinct traffic pattern. Koball et al. [23] proposed an unsupervised ensemble approach using one-class classifiers, feature aggregation, PCA, clustering, and K-Means to identify devices without labeled data, showing reasonable accuracy. To reduce reliance on labeled data, Fan et al. [24] developed a semi-supervised model based on CNN and multi-task learning that leverages timing, volume, protocol, and TLS-related features to separate IoT from non-IoT devices, achieving high accuracy with only 5% labeled data.

Liu et al. [9] focused on packet direction and length sequences in flows and used a 1D-CNN to extract features, reporting accuracy, recall, precision, and F1-scores above 99%, though they could not compare results across datasets and noted difficulties with unbalanced data and smaller training sets. Similarly, Ortiz et al. [10] employed a stacked LSTM autoencoder on raw TCP packets to learn latent classes and then modeled each device via DBSCAN clustering, but behavior identification remains challenging for devices of the same model.

Beyond classical classifiers such as Random Forest and SVM, recent work has explored more advanced architectures for traffic and device type classification. Luo et al. [21] propose a two stage Transformer-based model with traffic diagnosis and ensemble inference, achieving up to 100% accuracy in device type identification on heterogeneous IoT traffic. Similarly, Li et al. [20] introduce the ALOC framework, which uses a one-class adversarially trained 1D-CNN denoising autoencoder trained only on normal SCADA traffic to detect attacks with very low false positive and false negative rates. These approaches illustrate the recent shift from traditional feature-based models towards more expressive deep and Transformer-based architectures for network traffic analysis.

### 2.2. IoT Network Traffic Analysis and Generation

This subsection provides a comparative analysis of studies on the generation of synthetic network traffic for IoT devices. It examines various methodologies to simulate realistic network traffic patterns, enabling the complete training and evaluation of the model. In addition to realism and coverage, recent work has also emphasized the importance of privacy aware data generation in critical infrastructures. For example, in the LLM domain, the HARDLLM framework [26] proposes a differentially private query-driven data selection strategy over public and synthetic corpora to reduce the exposure of sensitive mission data to centralized servers. Inspired by this line of research, similar privacy-preserving mechanisms combining synthetic traffic generation with differentially private selection or retrieval could be adapted to IoT network traffic scenarios and integrated with device identification pipelines in deployments where raw traces cannot be directly shared. There is also a growing body of work surveying the security and safety aspects of large language models (LLMs). Despite their remarkable capabilities and growing adoption, LLMs also raise significant security and privacy concerns. These issues have been examined in detail by Zhou et al. [27], who surveyed the security, misuse, and privacy risks associated with models such as ChatGPT and provide an overview of current threats and corresponding mitigation strategies. A detailed comparison of the existing IoT traffic generation studies, including their methodologies and effectiveness, is presented Table 2.

IoTGemini [28] is a framework designed to create synthetic traffic for IoT networks, which helps in training models for analyzing network behavior without using real data. It includes two main parts: one that models the functions and behaviors of IoT devices, and another that generates realistic traffic patterns using advanced techniques like a Packet Sequence Generative Adversarial Network (PS-GAN). This approach allows for more accurate and customizable traffic generation, addressing limitations of previous methods that could not adapt to the diverse functions of modern IoT devices.

In [15] the methodology involves implementing and evaluating two GAN architectures—one using a Conditional Tabular GAN (CTGAN) and the other using a Copula GAN—to generate both categorical and continuous network traffic features. These models incorporate data transformation techniques, such as the Yeo–Johnson power transform and Gaussian Copula transformation, to improve data distribution and enhance synthetic data quality. The advantages of this approach include the ability to generate high-fidelity traffic features, the potential for enhanced training datasets for security applications, and the demonstration that 85% of generated features could pass as real data without detection. However, disadvantages include computational limitations, as training the models requires significant processing power, challenges in accurately modeling encrypted traffic, and the risk of mode collapse where the GAN fails to generate diverse traffic patterns. Instead of relying on costly real device deployments, the paper [16] proposes a method to synthetically generate network traffic that closely mimics real IoT device behavior. The methodology combines an autoencoder with a Wasserstein Generative Adversarial Network (WGAN). First, the autoencoder learns a latent representation of real packet size sequences. Then, a WGAN is trained in the latent space to generate realistic latent vectors, which are decoded back into sequences of packet sizes corresponding to bidirectional flows. The model is evaluated using traffic data from a Google Home Mini device.

**Table 2:** Summary of GAN-based studies for IoT network traffic feature generation.

Ref	Dataset(s)	Use Case	Features Used	Approach Summary
[14]	UGR’16	Balance IDS training data	Flow-level: duration, ports, flags	MLP-based GAN trained per attack type; normalized input; improved recall and F1
[29]	UNSW-NB15, NSL-KDD, BoT-IoT	IDS training using synthetic data	Flow: bytes, packets, flags	Deep GAN with 4 dense layers; boxplot-based convergence; 10K samples/dataset
[16]	Google Home Mini	NIDS evasion, flow mimicry	Packet-size sequences (categorical)	Autoencoder + WGAN (C/GP); trained in latent space; >98% evasion success
[28]	IoT testbed + public data	High-fidelity IoT traffic generation	Packet + flow: payload, IAT, direction	PS-GAN with LSTM layers; FSM simulation; function-device behavior profiling
[15]	UNSW-NB15	Tabular synthetic feature generation	49 flow features: protocol, ports, durations	Compared CTGAN, CopulaGAN, Vanilla GAN; CopulaGAN best via SDV metrics

As previously discussed, the related work was divided into two subsections: First, the studies focused on device identification based on network traffic analysis, highlighting the extraction of distinctive patterns to classify different IoT devices accurately. The second subsection covers synthetic data generation, especially in the field of network traffic by GAN models with the goal of augmenting datasets and mitigating data scarcity. Based on this literature review, the following points can be highlighted:

- The existing device-identification studies only partially address the severe class imbalance present in multi-device IoT traffic, often relying on naturally imbalanced datasets or simple resampling without systematically tackling skewed class distributions;
- The current GAN-based network traffic generation methods focus on reproducing overall statistical distributions but rarely incorporate higher-level semantic constraints (such as protocol semantics, device roles, or traffic context), which limits the realism and behavioral consistency of synthetic IoT traffic;
- Many GAN-based augmentation studies evaluate synthetic data mainly through visual or distributional similarity to real traffic, without thoroughly assessing how adding synthetic samples to an imbalanced dataset impacts IoT device classification performance, particularly for minority classes;

Therefore, the study aims to bridge these gaps by addressing the class imbalance challenge, which is a common issue in network traffic datasets because of the variety of IoT devices on the same network and the variety of packet sizes sent from/to other devices, which cause a class imbalance. The work proposes using a WGAN enhanced with graph-based conditioning and LLMs to generate semantically and structurally consistent synthetic samples.

## 3. Materials and Methods

**Figure 2:**
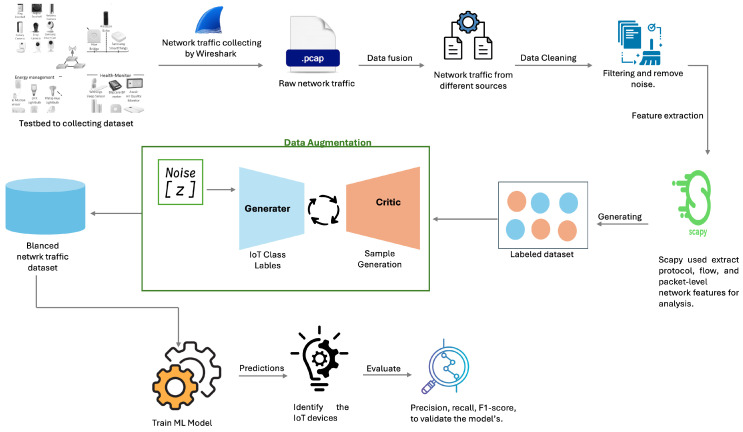
Architecture of the proposed work.

This section outlines the foundation of this study by detailing the dataset and the modeling framework. It first describes the data collection, feature extraction, and preprocessing steps, followed by an explanation of the modeling approach and experimental setup. Figure 2 illustrates the architecture of the proposed work. The subsequent subsections offer a comprehensive explanation of each component and its sequence.

### 3.1. Data Collection

The Internet of Things (IoT) dataset used in this study is the IoT Sentinel dataset [24]. It comprises network traffic from 27 IoT devices, including security cameras, smart plugs, water sensors, intelligent coffee appliances, door sensors, switches, and gateways. Each type of device was repeatedly setup for at least 20 repetitions, ensuring extensive data collection. The IoT Sentinel dataset is 65.6 MB in size and includes data from 27 distinct devices. In total, it contains 102,240 packets, distributed in 538 raw PCAP files. Scapy 2.6.1 (open-source Python packet manipulation library) was used to process the PCAP files and extract features from the network traffic and label them by device name. Each record represents a traffic instance described by numerical and categorical features.The extracted features include only header level traffic information (e.g., packet size, flag fields, ports, and DNS/TCP/UDP metadata), and do not store any payload content, user identifiers, or raw IP addresses. The dataset includes a label column indicating the device type or class, with multiple device categories (e.g., cameras, sensors, smart plugs, etc.). These labels were used to train both the feature constraint extractor and the WGAN model for class-conditional data generation.

#### 3.1.1. Network Features Analysis and Selections

**Figure 3:**
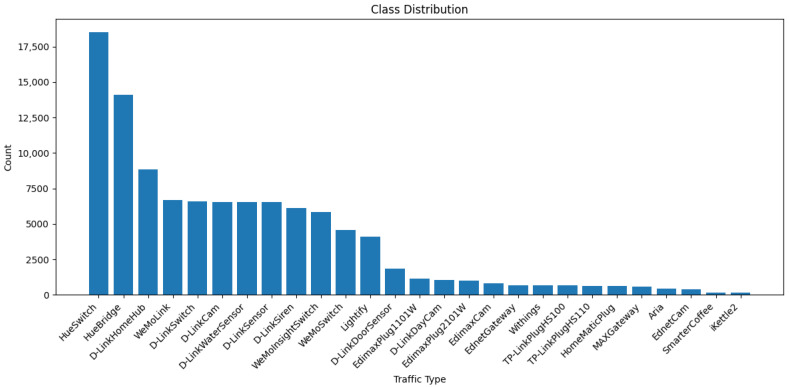
Class distribution of IoT device traffic types.

The dataset used in this study is designed for IoT device identification by analyzing the behavior of network packets without relying on IP addresses or payload data [30]. Instead, it focuses on packet header features extracted from network and transport layer protocols such as TCP, UDP, and IP, including attributes like packet size, protocol type, IP flags, TCP flags, source and destination ports, and Shannon entropy [31]. By leveraging these features, the dataset enables the classification of IoT devices based on their com-munication patterns, providing a generalizable approach applicable to various device types. The feature extraction process was conducted using a combination of advanced packet analysis tools, Python libraries, and network security frameworks to parse, analyze, and extract meaningful network features from PCAP files. The primary tools utilized in this study include Wireshark 4.6.2 and TShark 4.6.2 (Wireshark Foundation, Wilmington, DE, USA), PyShark 0.6, Scapy 2.6.1, Python 3.9 (Python Software Foundation, Wilmington, DE, USA), and the open-source data analysis libraries Pandas 2.2.2 and NumPy 2.0.2. Wireshark, a widely used network protocol analyzer, enabled deep inspection of network traffic, while TShark, its command-line counterpart, was employed to extract protocol-specific fields and generate structured output from PCAP files. The Scapy library in Python was instrumental in reading and parsing packet data, facilitating the extraction of detailed features. Furthermore, Pandas was employed for organizing and processing the extracted data into structured CSV files, while NumPy assisted in numerical computations and statistical feature transformations. Before developing our model, EDA was performed using Python to better understand numerical and categorical features, and data distributions and relationships. The visualizations such as bar chart for the categorical feature presented in Figure 3 illustrates the class distribution of the categorical variable “Traffic Type,” clearly highlighting the frequency of occurrences across various IoT device categories. This visualization aids in understanding class balance and potential data imbalance issues.

**Figure 4:**
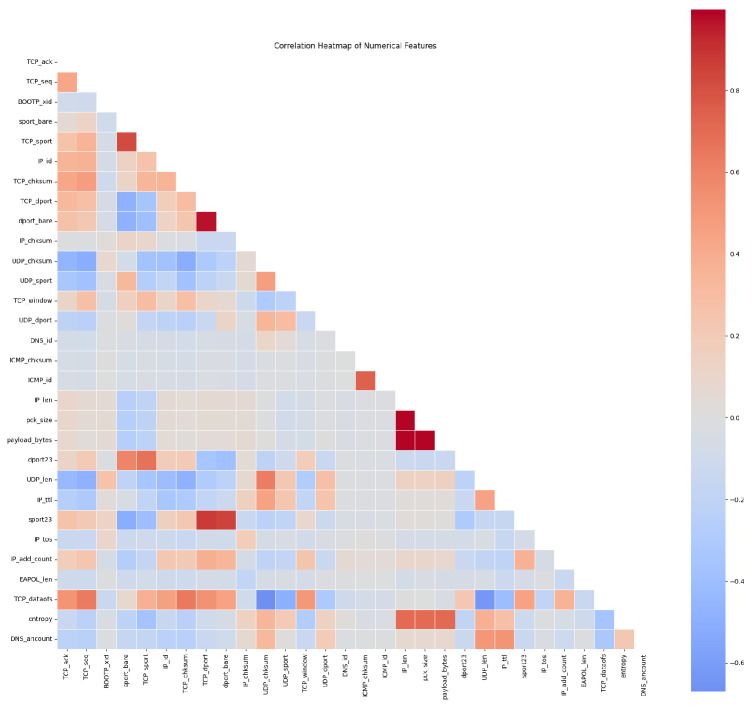
Correlation heatmap of numerical features.

As part of the data preprocessing, we computed the Pearson correlation matrix of the numerical features and visualized it as a heatmap in Figure 4. The plot reveals clear groups of strongly correlated variables, for example, length- and size-related features (p_len, pkt_size, payload_bytes, UDP_len), and features that are only weakly correlated with the rest. This indicates that the IoT traffic exhibits non-trivial dependencies between header and size features, and that some variables are partially redundant. These observations are used to better understand the structure of the dataset, guide subsequent feature analysis, and motivate our use of a feature relationship graph and graph-based conditioning in the proposed WGAN so that the generated traffic preserves these dependencies.

**Figure 5:**
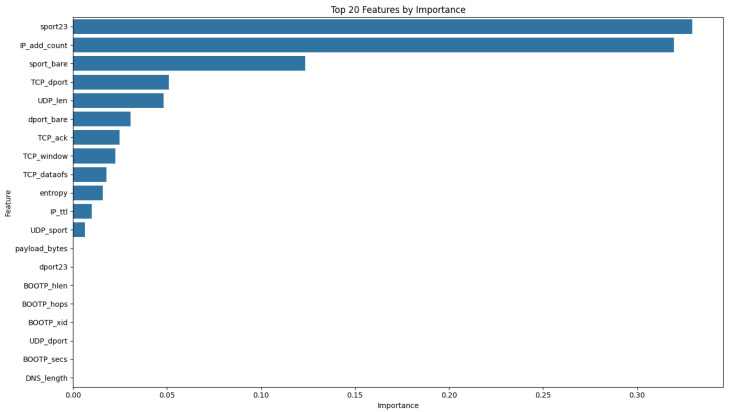
Feature importance using Random Forest.

Figure 5 illustrates the top 20 features. Table 3 provides a comprehensive summary of top 20 feature in the dataset, including its name, a detailed description, data type, and the rationale behind its classification. The information is intended to clarify the role of each feature in the dataset and support the subsequent data preprocessing and analysis steps.

#### 3.1.2. Preprocessing

In the preprocessing phase, we employ a systematic and adaptable pipeline to enhance the reliability and consistency of feature engineering. The methodology dynamically identifies numerical and categorical features using a combination of predefined configurations and data driven checks, ensuring robust feature classification even in the presence of unexpected data types. Missing values are imputed using the arithmetic mean for numerical features and the mode for categorical features, supporting data completeness while limiting distortions. Numerical attributes are standardized via StandardScaler class from scikit-learn 1.6.1, and categorical attributes are encoded in real time. The SafeEncoder first converts all categorical values to textual form to reduce inconsistencies before encoding. Class distributions are preserved through a stratified train, validation, and test split, which mitigates the impact of class imbalance. In addition, a fallback mechanism safeguards critical numerical features in case of transformation failures, thereby maintaining data integrity. While the primary goal is to provide a structured, flexible, and error-tolerant preprocessing workflow, the approach can be further extended to better handle complex categorical hierarchies and task-specific feature selection.

### 3.2. Methodolgy

In this section, we describe the proposed methodology for device type identification based on imbalanced IoT network traffic. The pipeline proceeds in three main stages: First, we construct a feature relationship network graph from the raw traffic data to capture statistical and structural dependencies between features. Second, we use a LLM to extract semantic constraints from this graph and integrate them into a WGAN-based generator to synthesize additional, device-specific traffic samples. As a baseline, we also train a CTGAN model under the same feature configuration and sampling conditions, allowing a fair comparison between the proposed constraint-guided WGAN and a standard tabular GAN. Finally, we train and evaluate classification models on a merged dataset consisting of real traffic and WGAN-generated samples in order to assess the impact of the proposed generative strategy on device type identification performance.

#### 3.2.1. Network Graph Construction

We construct a feature relationship graph from the training data using three complementary dependency measures: Pearson correlation (linear associations), Spearman rank correlation (monotonic non-linear trends), and Mutual Information (general non-linear dependencies). When preprocessed features are available, we use the provided matrix; otherwise, we parse numeric columns from the raw data (coercing non-numeric values and imputing missing entries with zero). Constant or near-constant features (variance $\leq 10^{-10}$) are removed prior to analysis. For numerical stability, NaNs in correlation matrices are replaced with zeros, and MI is computed with a capped sample size (up to 5000 points) and then row normalized; the MI matrix is symmetrized.

The three measures are integrated into a composite relationship matrix

(1) R=0.4|P|+0.3|S|+0.3M

where *P*, *S*, and *M* are the Pearson, Spearman, and MI matrices, respectively. Domain-informed grouping (e.g., size, time, ID, and port related attributes) is used to strengthen within-group links by enforcing a minimum edge weight of 0.2. Edges are retained for pairs with $R_{ij}>0.05$, yielding a weighted, undirected graph G=(V,E) whose nodes are features and edge weights encode relationship strength. If no edges satisfy the statistical criterion, a small set of domain-expected links (e.g., packet size payload, TCP sequence acknowledgment, and source destination ports) is injected to ensure minimal connectivity. Node importance is estimated by summing degree, betweenness, and eigenvector centralities. The highest scoring features are reported, and graph labels default to the most connected nodes when the graph is dense. We also export the full relationship matrix, the adjacency matrix of *G*, and visual diagnostics (graph layout and a heatmap over the most connected subset) for interpretation.

#### 3.2.2. WGAN Synthetic Dataset Generation with Network Graph and LLM

The experiment is an innovative approach to synthetic data generation that integrates network graph conditioning and LLMs within WGAN. The aim is to improve training stability and the quality of synthetic data generation, achieve a balanced dataset, and produce realistic network traffic data suitable for cybersecurity research, anomaly detection, and AI model training. The Wasserstein distance is used in the WGAN to compare distributions for synthetic data generation, while graph conditioning helps the generator capture real-world relationships between network traffic features. LLM integration ensures that the generated traffic complies with cybersecurity regulations, such as valid protocol behavior. By incorporating graph-based feature relationships extracted from the network graph and leveraging LLM-derived constraints, the model ensures that the generated synthetic data maintains structural integrity and adheres to domain-specific rules. An essential component of this approach is the graph conditioning mechanism, which supplements the process of generating synthetic data with structural links between attributes. The WGAN’s generator uses an adjacency matrix extracted from the feature relationship graph produced in the previous step to add latent dependencies. In order to guarantee that the synthetic samples display genuine feature interactions, the adjacency matrix encodes both statistical correlations (such as Pearson and Spearman correlation) and domain-specific associations. Additionally, the generator is conditioned using the key characteristics found in the network by centrality measurements, which enables the model to concentrate on maintaining high-impact linkages in the data. By imposing protocol-specific restrictions, the incorporation of LLM improves the generation process and guarantees that synthetic data complies with logical and regulatory requirements. Structured domain information, such as numerical constraints, feature correlations, and protocol rules unique to each data category, is obtained by querying GPT-4 (OpenAI, San Francisco, CA, USA). This improves WGAN’s realism and simulation usability by allowing it to generate data that more closely resembles actual patterns. In order to guarantee that the outputs stay within the proper domain range, these constraints are dynamically applied to the created samples. In order to improve the trustworthiness of the created data, LLM is also utilized in post generation data validation, where it evaluates sample quality, provides validity scores, and makes recommendations for enhancements based on expert domain.

In this approach, the generator is not only trained to deceive the discriminator but also to satisfy a set of semantic rules derived from the LLM. As outlined in Algorithm 1, the critic is updated multiple times per iteration using the standard objective, and the generator is then updated with an augmented loss that includes a semantic constraint penalty. This procedure encourages the generator to produce samples that are both adversarially plausible and compliant with class-specific numeric ranges and categorical vocabularies defined by the domain constraints. The LLM guidance is operationalized through a loss-penalty mechanism, where constraints are represented as numerical bounds or discrete sets in a JSON structure and directly incorporated into the training loss, rather than through reinforcement learning or textual scoring. Furthermore, the process is dynamic: every *K* epochs, generated samples are summarized and re-evaluated using prompt-based LLM feedback, which adaptively updates the penalty weight $\lambda_{c}$ and the importance of specific features. This iterative feedback loop allows the generator to progressively refine its outputs, ensuring that the synthesized data are both statistically realistic and semantically coherent.
**Algorithm 1** Main Training Loop: WGAN-GP with Semantic Constraints**Input:** Data *X*, labels *y*, constraints C, epochs *E*, batch size *B*, latent dim *z*, learning rate η, critic steps $n_{\mathrm{critic}}$, GP weight $\lambda_{\mathrm{GP}}$, constraint weight $\lambda_{c}$**Output:** Trained Generator *G* and Discriminator (critic) *D*  1:Initialize $G:R^{z}\;\to \;R^{d}$ (Tanh output), $D:R^{d}\;\to \;R$; optimizers (Adam, $\beta_{1}=0.5,\beta_{2}=0.999$)  2:Preprocess *X* with class-wise scaling/encodings; build DataLoader of (x,y)  3:**for** e←1 to *E* **do**  4:    **for** each batch $\left(x,y_{b}\right)$ of size *B* **do**  5:        **Critic update**  6:        Sample z∼N(0,I); $\tilde{x}\leftarrow G\left(z\right)$                                    ▷ no grad for *G* at critic step  7:        $L_{D}\leftarrow E\left[D\left(\tilde{x}\right)\right]-E\left[D\left(x\right)\right]+\lambda_{\mathrm{GP}}\cdot \mathrm{GP}\left(x,\tilde{x}\right)$  8:        Update *D* by descending $\nabla L_{D}$  9:        **if** (batch_idx mod $n_{\mathrm{critic}}=0$) **then**10:           Generator update with semantic constraints11:           Sample z∼N(0,I); $\hat{x}\leftarrow G\left(z\right)$12:           Compute constraint penalty $\Psi (\hat{x},C;y_{b})$              ▷ Decode $\hat{x}$ per class, score violations vs. C13:           $L_{G}\leftarrow -\;E\left[D\left(\hat{x}\right)\right]+\lambda_{c}\cdot \Psi \left(\hat{x},C;y_{b}\right)$14:           Update *G* by descending $\nabla L_{G}$15:        **end if**16:    **end for**17:    (Optional) Step LR schedulers; log $\left\{L_{D},L_{G}\right\}$; save curves and intermediate samples18:**end for**19:**return** G,D

#### 3.2.3. CTGAN

To isolate the effect of the generator family, we include CTGAN as a baseline trained under exactly the same pipeline used for WGAN. Preprocessing steps, the feature set, the semantic constraint, batch size, and the validation protocol are kept identical so that differences can be attributed to the model architecture rather than training conditions. CTGAN follows a conditional generator discriminator design suitable for mixed data; categorical variables are embedded and concatenated with continuous features. The model receives the same conditioning used by WGAN, combining the domain/task conditions with the constraint token vector. The discriminator is conditioned in the same way. We integrate the same constraint mechanism used in our WGAN setup: during training, generated batches are checked against data-derived constraints’ empirical value ranges, and valid category sets estimated on the training split, together with cross feature dependencies inferred from the feature relationship graph and violations, contribute a penalty that encourages valid samples. During sampling, a light rejection step removes samples that exceed a small violation tolerance.

#### 3.2.4. Machine Learning Modeling

This section presents the methodology used to train and evaluate multiple machine learning models that assess the effectiveness of the proposed data set (both real and synthetic samples generated by WGAN). The dataset needs to be balanced after generating the synthetic samples using WGAN; therefore, the ML model must learn from all classes equally. This experiment features a wide range of machine learning models, each one fine-tuned via deliberately chosen hyperparameters to ensure balanced performance between predictive accuracy, generalization, and computational efficiency. The Random Forest Classifier is an ensemble learning technique that develops multiple decision trees and then takes an aggregate of these outputs to make the classification more stable. The model will provide an equilibrium between efficiency and the amount of learning that can take place if set to 100 estimators (n_estimators = 100) and allow unending growth of trees (max_depth = None). Since a minimum of 2 samples are needed to split, computation is more efficient that way (min_samples_split = 2), and this also further decreases training time by implementing parallelism, and all the CPU cores are utilized (n_jobs = −1).

The SVM classifier model is included so that we can leverage its kernel-based learning since it is very good in high-dimensional feature spaces. The model has an RBF kernel to be able to express non-linear relationships in features, all while keeping the computation efficient. The regularization parameter is a compromise between maximizing the margin and minimizing the misclassification so that it can be of good use for estimation. We also have to turn on probability estimates to make it easy to interpret the model for classification tasks. A neural network-based method for capturing complex feature interactions is represented by the Multi-Layer Perceptron (MLP), the final model. A total of 100 neurons make up its single hidden layer (hidden_layer_sizes = (100)), and the ReLU activation function (activation = ‘relu’) is used to account for non-linearity. The Adam optimizer (solver = ‘adam’), which adaptively modifies learning rates to improve convergence speed, is used to optimize the model. To prevent overfitting, a regularization parameter (alpha = 0.0001) is used, and 200 training iterations (max_iter = 200) guarantee adequate learning without putting an undue strain on the computer.

## 4. Experiments and Results

The experiments were conducted to evaluate the effectiveness of the proposed constraint-guided WGAN framework, which integrates network graph-based feature relationships and LLM-derived semantic constraints for synthetic network traffic generation. The experimental workflow follows a multi stage pipeline that begins with the extraction of raw features from captured PCAP files and proceeds through preprocessing, graph construction, constraint extraction, and WGAN training. The process starts with raw network traffic data, where Scapy is used to extract flow level and packet level statistical features. The data preprocessing stage detects numerical and categorical attributes, scales values to the range [−1, 1] [−1, 1], and encodes categorical features for model compatibility. Next, the network graph construction module computes Pearson, Spearman, and mutual information correlations among features to build a feature relationship graph, which helps identify relevant dependencies. A constraint extraction step is then performed using GPT-4, producing a structured JSON file that defines valid feature ranges, allowed categorical values, and correlation patterns for each device class. These constraints are subsequently integrated into the constraint-guided WGAN training, where the generator is regularized through penalty terms that enforce semantic and statistical consistency. Finally, the trained generator produces a synthetic dataset that is inverse transformed back to the original feature scale and balanced across device classes for machine learning evaluation.

The following subsection provides a more detailed explanation of the synthetic data generation process, including the integration of the network graph, LLM-based constraint extraction, and WGAN training procedure.

### 4.1. Network Graph Construction Results

**Figure 6:**
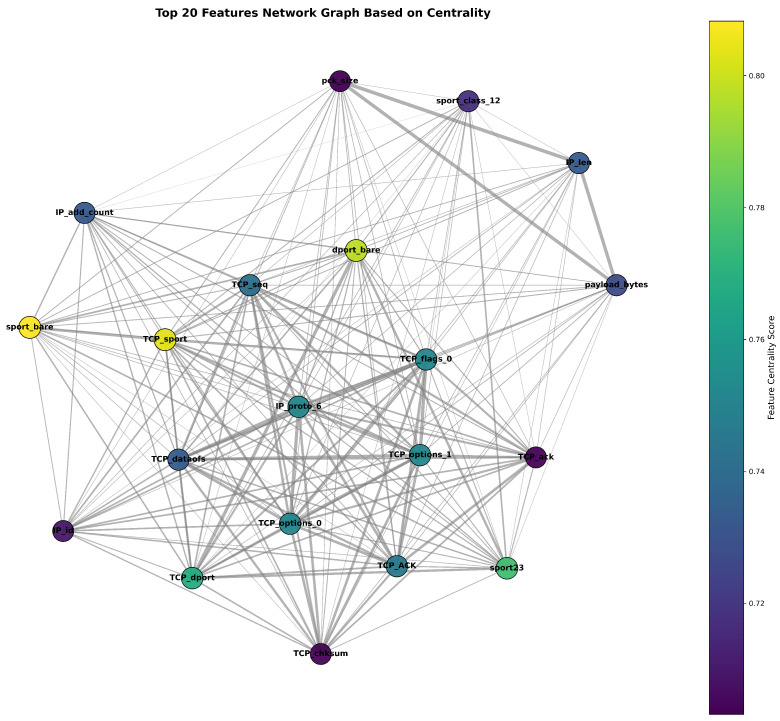
Feature correlation network.

As we mentioned before, the network graph visualizes the pairwise relationships among the extracted features as a graph. Figure 6 shows the 20 main central features extracted from our IoT traffic dataset, where node size and color intensity encode the combined centrality score of each feature, and the edge thickness reflects the strength of pairwise correlations. The topology not only reveals which features are most influential and tightly coupled but also guides our feature selection: we prioritize nodes with both high centrality and strong inter-feature correlations to construct more robust classification models.

### 4.2. Synthetic Dataset Generation Model with Network Graph and LLM

**Figure 7:**
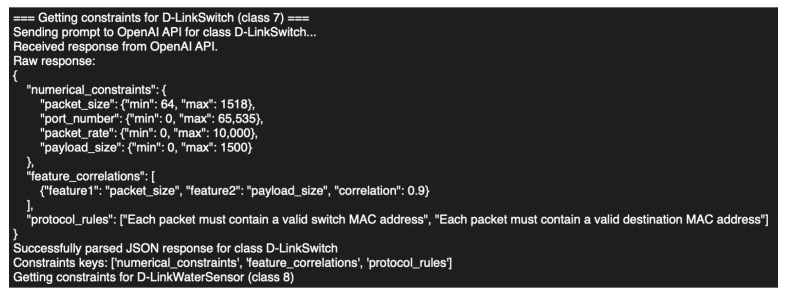
Example of constraint extraction for D-Link Switch (Class 7).

We utilized a data dimension of 97 features and 27 device classes. The adjacency matrix, representing feature relationships, had a size of (97, 97), ensuring the preservation of statistical and domain-specific dependencies during data generation. The constraints derived from the LLM model were extracted for each device class, ensuring that the generated samples adhere to protocol-specific rules, feature correlations, and numerical constraints. Figure 7 illustrates example of extraction of numerical, correlational, and protocol constraints for the D-LinkSwitch class. After sending the prompt, our parser automatically identified and validated three sections (numerical_constraints, feature_correlations, and protocol_rules), which we fed into the downstream traffic-generation module.

#### 4.2.1. WGAN Model (Constraint-Guided)

The WGAN model was trained over 100 epochs, and the critic network was updated five times per generator update (CRITIC_STEPS = 5) to maintain the balance between discriminator and generator learning. The loss of the critic and the loss of the generator was monitored throughout the training to evaluate convergence. The key training parameters of the WGAN model are presented in Table 4.

**Figure 8:**
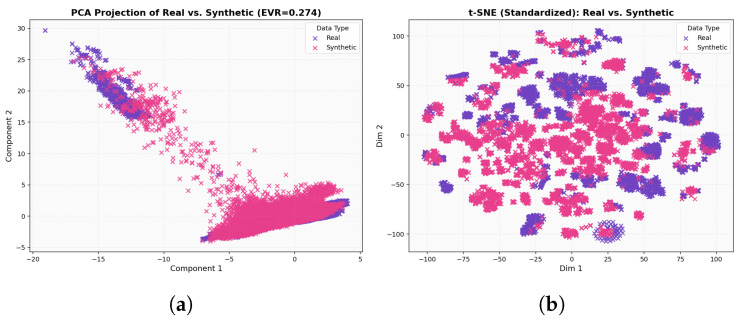
(**a**) Two dimensional PCA of real and WGAN-generated samples; (**b**) t-SNE embedding of real and WGAN-generated samples.

In the 2D PCA diagram Figure 8a, the real and generated samples show substantial overlap, appearing as two largely congruent point clouds with only minor deformations. The generated distribution is slightly elongated, effectively stretching to span low-density gaps and cover implanted (rare) regions, but without introducing pronounced shifts or artifacts. Taken together, this visualization indicates that the WGAN has reproduced the global variance (covariance) structure of the data with high fidelity, capturing the principal components and overall manifold while exhibiting only small calibration differences.

**Figure 9:**
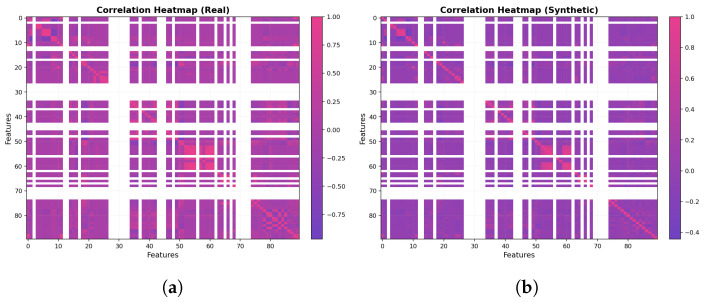
(**a**) Correlation heatmap for the real dataset; (**b**) correlation heatmap for the WGAN-generated dataset showing closely matched block-wise dependencies and correlation strengths.

**Figure 10:**
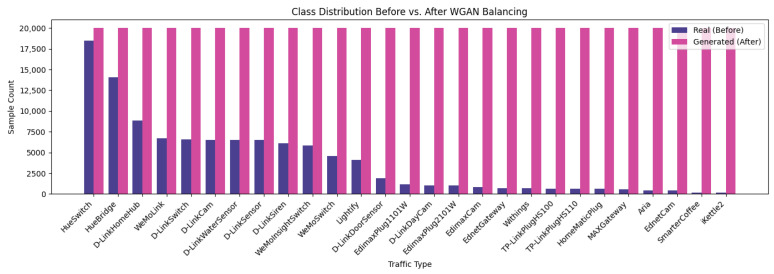
Class distribution before vs. after WGAN balancing.

For the t-SNE in Figure 8b, the real and generated samples largely occupy the same regions, forming grouped clusters that align across both datasets. The synthetic clusters occasionally appear slightly denser or sparser than their real counterparts, reflecting intentional coverage of class imbalance, yet the overall map reads as a homogeneous mixture rather than separated swaths. This pattern indicates that, at the local-neighborhood scale emphasized by t-SNE, the generated data has effectively captured the same underlying manifold as the real data. In the correlation heatmaps Figure 9, the real and generated matrices appear nearly indistinguishable: strong and weak correlations recur in the same blocks, and the fine-grained patterns are closely mirrored across features. This close visual agreement indicates that the WGAN has effectively captured the covariance and inter-feature dependency structure, reproducing both the dominant blocks and the subtle relationships with near-perfect fidelity. As shown in Figure 10, the real (purple) distribution is highly skewed, whereas the WGAN-balanced set (pink) is approximately uniform across classes.

#### 4.2.2. CTGAN (Constraint-Guided) Baseline

To test whether our findings depend on the generator family, we evaluated CTGAN under the same pipeline used for WGAN. All constraints were extracted from the training data using an LLM: the LLM consumed feature statistics and network graph relationships and outputted a structured rule set (empirical value ranges, valid category sets, formatting/type checks, and cross-feature relations). These data-derived constraints were saved to a JSON specification and kept fixed for all models. CTGAN uses the same condition vector as WGAN (domain/task conditions plus constraint tokens), and the same preprocessing, train/validation split, batch size, learning rates, and optimizer. We also applied the same enforcement: violations during training increase the generator’s objective, and a light rejection step filtered invalid samples at inference using the same tolerance. Table 5 summarizes the primary CTGAN settings.

**Figure 11:**
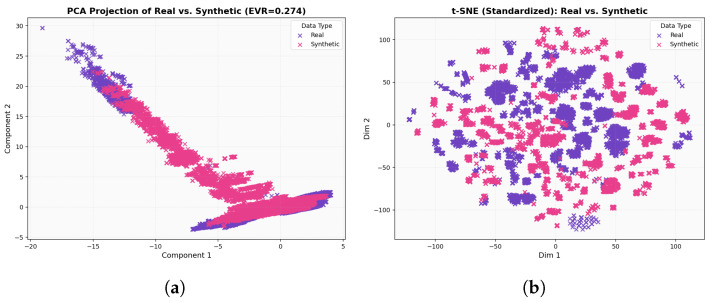
(**a**) Two dimensional PCA of real and CTGAN-generated samples; (**b**) t-SNE embedding of real and CTGAN-generated samples.

**Figure 12:**
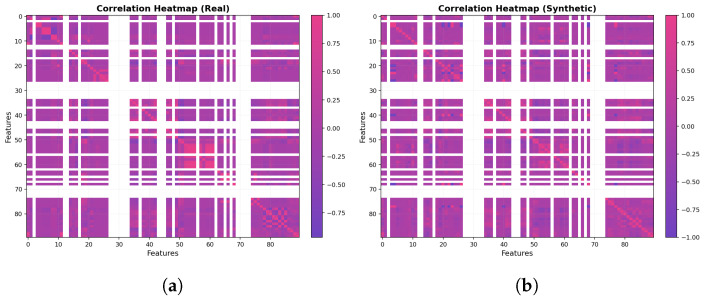
(**a**) Correlation heatmap for the real dataset; (**b**) correlation heatmap for the CTGAN-generated dataset.

For the result of CTGAN, the two dimensional PCA projection in Figure 11a shows that real and synthetic samples occupy a broadly similar region of the feature space, with both clouds aligned along the same dominant manifold. However, the synthetic distribution appears slightly more compact and smoothed, with reduced spread in the extreme regions, indicating that CTGAN captures the main axes of variance but underestimates some of the tail behavior present in the real data. A complementary t-SNE visualization in Figure 11b confirms this pattern at the local scale: synthetic points are interleaved with real samples within most clusters, but some clusters are more diffuse or partially merged, suggesting a tendency to regularize highly sparse or imbalanced regions. The correlation heatmaps in Figure 12b further illustrate that CTGAN reproduces the overall block structure of inter-feature dependencies, with strong and weak correlations recurring in the same groups of features, while a subset of blocks exhibits attenuated correlation magnitudes. Taken together, these qualitative analyses indicate that CTGAN provides a reasonable but slightly over-smoothed approximation of the real traffic distribution, capturing the dominant covariance structure while losing some of the fine grained variability that is better preserved by the WGAN model.

#### 4.2.3. WGAN vs. CTGAN

To better understand the behavior of the two generative models, we qualitatively compared WGAN and CTGAN along three complementary views: global variance structure (PCA), local neighborhood structure (t-SNE), and inter-feature dependencies (correlation heatmaps). Both models are able to place synthetic samples in the same broad region of the feature space as the real traffic, indicating that they capture the dominant modes of variation. However, consistent differences emerge when inspecting the fine-grained alignment between the real and generated distributions.

For the WGAN, the PCA projection shows a strong overlap between the real and synthetic samples along the main manifold, with only minor elongations at the tails, while the t-SNE plot reveals interleaved clusters rather than separated real–synthetic islands. The corresponding correlation heatmaps for WGAN are also closely matched to the real matrix, reproducing the block structure and most of the strong/weak correlations. In contrast, CTGAN produces a slightly more compact and smoothed distribution in PCA space, with underestimation of some extreme regions, and t-SNE clusters that are sometimes more diffuse or partially merged. Its correlation heatmaps recover the main blocks of dependencies but tend to attenuate some of the correlation magnitudes, particularly in sparse or highly imbalanced regions.

Overall, these observations suggest that while CTGAN provides a reasonable approximation of the real traffic and is effective at regularizing very sparse regions, WGAN offers a closer match to both the global manifold and the local cluster structure, and more faithfully preserves inter-feature dependencies. For this reason, we adopt WGAN as the primary generator in the remainder of our experiments, and use CTGAN mainly as a comparative baseline.

### 4.3. Machine Learning Performance

The evaluation was conducted on the machine learning models mentioned in the previous section using the preprocessed dataset. The original real traffic dataset was first partitioned into three disjoint subsets using a stratified split on the device label, yielding 67,734 samples for training, 16,934 for validation, and 21,167 for testing, as reported in Table 6. Stratification ensures that the real data for each device type are proportionally represented in all three subsets and reduces the risk of bias toward any particular class.

After this initial split, we employed the WGAN-generated samples to balance the label distribution within each subset. For every device class in the training, validation, and test sets, synthetic flows were added up to a fixed per-class target so that all the classes are represented with (approximately) equal frequency. This procedure has three benefits: (i) each subset contains a mixture of genuine and generated traffic for every device type; (ii) the class distribution within each subset is effectively balanced; and (iii) the separation between training, validation, and test sets is preserved, preventing information leakage. The balanced training and validation subsets are then used to fit and tune the machine learning models, while the balanced test subset is used exclusively for final performance evaluation.

The performance of each model was assessed using accuracy, precision, recall, and F1-score, along with computational efficiency in terms of training time and memory consumption. Table 7 summarizes the overall test performance of the models.

On the WGAN-balanced dataset, Random Forest achieved the best overall performance (accuracy (=0.9409), precision (=0.9418), recall (=0.9409), F1 (=0.9402)). It also trained the fastest ((15.37) s) but consumed the most memory ((2558.66) MB). Neural network ranked second (accuracy (=0.9083), precision (=0.9093), recall (=0.9083), F1 (=0.9074)) with a training time of (139.41) s and memory use of (443.39) MB. SVM recorded the lowest scores (accuracy (=0.8899), precision (=0.8903), recall (=0.8899), F1 (=0.8884)), required the longest training time ((26,513.04) s), and used the least memory ((10.72) MB).

In addition, the SMOTE technique was applied to the data set to address class imbalance, followed by classification using various machine learning models. The performance of each model was comprehensively evaluated using multiple metrics, including precision, precision, recall, F1 score, training time, and memory usage. The results are summarized and compared in the corresponding evaluation Table 8, which provides a comparative analysis of three machine learning models applied to a SMOTE-balanced data set.

On the SMOTE-balanced dataset, Random Forest achieved the best results (accuracy (=0.9246), precision (=0.9394), recall (=0.9246), F1 (=0.9283)). It also trained the fastest (88.60) s) but used the most memory (177.64) MB). Neural network lagged behind (accuracy (=0.3492), precision (=0.4852), recall (=0.3492), F1 (=0.2842)) with a training time of (142.17) s and memory of (82.13) MB. SVM performed worst (accuracy (=0.0618), precision (=0.0038), recall (=0.0618), F1 (=0.072)), took the longest to train ((1297.76) s), and used (92.10) MB of memory.

**Figure 13:**
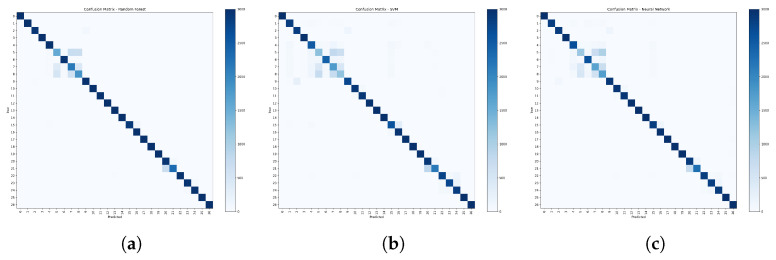
(**a**) Random Forest, (**b**) SVM, and (**c**) neural network confusion matrices.

The confusion matrices in Figure 13 show strong main diagonal high accuracy with most errors concentrated among a small family of look alike devices chiefly D-LinkSensor, D-LinkWaterSensor, D-LinkSwitch, and the sibling plugs TP-LinkPlugHS110 and TP-LinkPlugHS100 which are frequently confused with one another. Random Forest yields the cleanest diagonal and the narrowest spill within this D-Link/TP-Link cluster; the neural network is close but exhibits slightly more bleed, while SVM shows the broadest cross-class confusion. Outside this cluster, several classes are classified almost perfectly, notably MAXGateway, HomeMaticPlug, Withings, iKettle2, and D-LinkDoorSensor. This pattern aligns with the aggregate metrics (Random Forest best on accuracy/F1) and suggests that remaining errors stem from intra brand similarities, motivating a hierarchical family first classifier or richer discriminative features to separate the D-Link/TP-Link subgroup.

## 5. Discussion

The main objective of this work is to investigate whether a graph and LLM-guided WGAN can provide high-quality synthetic IoT traffic for addressing severe class imbalance in device identification. The results across both qualitative and quantitative evaluations indicate that the proposed framework does not merely increase the number of minority samples, but also preserves essential structural properties of the real traffic in a way that is beneficial for downstream classifiers.

### 5.1. Qualitative Behavior of WGAN vs. CTGAN

The qualitative comparison between the proposed WGAN configuration and a CTGAN baseline, using two dimensional PCA projections, t-SNE embeddings, and feature feature correlation heatmaps, shows that both generators are capable of producing plausible synthetic flows that occupy the same broad region of the feature space as the real data. However, the WGAN exhibits a visibly tighter alignment with the real traffic: in PCA space, real and WGAN-generated points largely overlap along the main manifold; in the t-SNE plots, clusters associated with different device types appear interleaved rather than separated into real versus synthetic islands; and in the correlation heatmaps, the WGAN closely reproduces the block structure and relative strength of inter-feature dependencies. By contrast, the CTGAN baseline tends to produce a smoother and more compact distribution, slightly under representing extreme regions and weakening some correlations, especially for device types with very few real samples. These qualitative observations motivate the choice of the WGAN as the primary generator for constructing the balanced datasets used in the subsequent classification experiments, while CTGAN is retained as a secondary qualitative baseline.

### 5.2. Impact of WGAN-Based Balancing on Classification Performance

The classification results on balanced datasets highlight the effectiveness of the proposed WGAN-based balancing strategy compared with a conventional oversampling method such as SMOTE. When training on WGAN-balanced data, the evaluated machine learning models consistently achieve higher macro-F1 and balanced accuracy than when trained on SMOTE-balanced data, confirming that the quality and structure of synthetic flows are at least as important as their quantity. The WGAN-generated samples provide richer information about minority device types, enabling the classifiers to better separate classes that are severely under represented in the original traffic.

Across all the regimes, Random Forest emerges as the most effective classifier, delivering the highest overall performance and the most stable behavior across metrics. The neural network remains competitive, particularly on the WGAN-balanced data, while SVM systematically lags behind on this high-dimensional, multi-class device-identification task. These trends are consistent with the intuition that ensemble and deep models are better able to exploit the nuanced, graph and constraint-preserving structure present in the WGAN-generated flows, whereas SVM is more sensitive to residual overlap and class complexity.

From a computational perspective, the WGAN-balanced regime is also attractive in practice. For example, when trained on WGAN-generated balanced data, the Random Forest model achieves superior predictive performance while requiring substantially less training time than on SMOTE-balanced data (15.37 s versus 88.60 s), which is an important consideration for large-scale or frequently retrained deployments. Although the WGAN-based setting incurs a higher memory footprint for Random Forest (2558.66 MB versus 177.64 MB), this overhead is often acceptable in modern computing environments given the corresponding gains in accuracy and robustness. Overall, these results indicate that the combination of a feature relationship graph, LLM-guided semantic constraints, and a WGAN backbone yields a synthetic data generation mechanism that not only corrects class imbalance, but also enhances the discriminative power and efficiency of standard machine learning models for IoT device identification.

### 5.3. Limitations and Future Work

Although the proposed framework already shows clear advantages over SMOTE and a CTGAN baseline, several aspects remain open for further strengthening: First, we evaluate the current system on a stationary traffic snapshot and therefore do not explicitly measure long term data drift, even though the design naturally allows re-estimating the feature relationship graph and recomputing the LLM-derived constraints on new traffic windows, followed by retraining the WGAN, to adapt to evolving behavior in future deployments. Second, while the LLM-guided constraints are derived only from aggregated feature descriptions and never access raw payloads, integrating formal privacy-preserving and robust LLM techniques would provide additional assurance in security-critical settings. Third, it will be important to analyse how the generated traffic behaves under adversarial conditions, where an attacker might attempt to exploit synthetic samples to evade detection or poison downstream models. Exploring these directions will further enhance the reliability and impact of the proposed approach in AI-assisted cybersecurity and imbalanced network data analysis.

## 6. Conclusions

In conclusion, the paper highlights the effectiveness of addressing class imbalance in the IoT network traffic that was collected from a variety of devices operating in a smart environment. The WGAN with a network graph and LLM was combined to balance the class in the data set. After that, we analyzed the performance of three machine learning models and compared them with data that were balanced using the SMOTE technique. It was demonstrated that using WGAN based on a network graph and LLM for synthetic data generation has a significant positive impact on the contextual quality and semantic accuracy of the generated sample, which directly impacts the performance of the trained models. Future research is recommended to explore ways to customize LLM embeddings to suit domain-specific synthetic data generation, as well as evaluate their potential across a wide range of classification tasks and complex datasets.

## Figures and Tables

**Table 3:** Feature details and classification rationale.

Feature Name	Description	Type	Reason for Classification
IP_add_count	Number of IP addresses in the packet	Numerical	Represents a count (discrete but treated as numerical)
IP_chksum	Checksum of the IP header	Numerical	Continuous quantity (checksum value)
sport_bare	Source port value without classification	Numerical	Continuous quantity (port number)
IP_id	Identification field in IP header for reassembly	Numerical	Unique identifier, treated as numerical
TCP_sport	Source port in TCP header	Numerical	Continuous quantity (port number)
TCP_window	Window size in TCP header	Numerical	Continuous quantity (window size)
TCP_dport	Destination port in TCP header	Numerical	Continuous quantity (port number)
TCP_seq	Sequence number in TCP header	Numerical	Continuous quantity (sequence number)
dport_bare	Destination port value without classification	Numerical	Continuous quantity (port number)
entropy	Entropy of the payload	Numerical	Continuous quantity (entropy value)
payload_bytes	Size of the payload in bytes	Numerical	Continuous quantity (payload size)
pck_size	Size of the packet in bytes	Numerical	Continuous quantity (packet size)
TCP_ack	Acknowledgment number in TCP header	Numerical	Continuous quantity (acknowledgment number)
dport_class	Class of destination port	Categorical	Represents distinct port classes
IP_len	Total length of the IP packet	Numerical	Continuous quantity (IP packet size)
sport23	Source port value (if applicable)	Numerical	Continuous quantity (port number)
TCP_dataofs	Data offset in TCP header	Numerical	Continuous quantity (offset value)
sport_class	Class of source port	Categorical	Represents distinct port classes
UDP_sport	Source port in UDP header	Numerical	Continuous quantity (port number)

**Table 4:** Key model parameters and training settings for WGAN.

Parameter	Value/Setting
Model Type	WGAN (Constraint-Guided)
Input Features	97
Latent Dimension (z)	100
Epochs	100
Batch Size	128
Learning Rate	0.0002
Optimizer	Adam ($\beta_{1}=0.5$, $\beta_{2}=0.999$)

**Table 5:** Key model parameters and training settings for CTGAN.

Parameter	Value/Setting
Model Type	CTGAN (Constraint-Guided)
Input Features	97
Latent Dimension (z)	100
Epochs	100
Batch Size	128
Learning Rate	0.0002
Optimizer	Adam ($\beta_{1}=0.5$, $\beta_{2}=0.999$)

**Table 6:** Dataset overview: number of samples per category.

Data Set	Number of Samples
Training Set	67,734 samples
Validation Set	16,934 samples
Test Set	21,167 samples

**Table 7:** Performance of machine learning models on WGAN-balanced dataset.

Model	Accuracy	Precision	Recall	F1 Score	Training Time	Memory (MB)
Random Forest	0.9409	0.9418	0.9409	0.9402	15.37s	2558.66
SVM	0.8899	0.8903	0.8899	0.8884	26,513.04	10.72
Neural Network	0.9083	0.9093	0.9083	0.9074	139.41	443.39

**Table 8:** Performance of machine learning models on SMOTE-balanced dataset.

Model	Accuracy	Precision	Recall	F1 Score	Training Time	Memory (MB)
Random Forest	0.9246	0.9394	0.9246	0.9283	88.60 s	177.64
SVM	0.0618	0.0038	0.0618	0.072	1297.76 s	92.10
Neural Network	0.3492	0.4852	0.3492	0.2842	142.17 s	82.13

## Data Availability

The datasets generated and analyzed during the current study are available from the corresponding author upon request.
